# MRI imaging findings in prune perineum syndrome: an extremely rare entity

**DOI:** 10.1259/bjrcr.20200153

**Published:** 2021-01-20

**Authors:** Isaac Torres de Carvalho Alves, Matheus Dorigatti Soldatelli, Sérgio Cavalheiro, Samir Sari Omar, Bruno Shigueo Yonekura Inada, Leonardo Furtado Freitas, Lázaro Faria do Amaral, Tauy Pereira Morimoto

**Affiliations:** 1 Department of Radiology, Hospital da Beneficência Portuguesa, São Paulo, Brazil; 2 Department of Neuroradiology, Hospital da Beneficência Portuguesa, São Paulo, Brazil; 3 Department of Pediatric Neurosurgery, Hospital Santa Catarina, São Paulo, Brazil; 4 Department of Neurosurgery, University Federal of São Paulo (UNIFESP), São Paulo, Brazil

## Abstract

We report a case of prune perineum syndrome, an extremely rare entity with only four cases reported to date, describing some typical clinical and radiologic features. We also present a newly associated imaging finding, the terminal ventricle’s cystic dilatation, and briefly discuss the differential diagnosis.

## Introduction

Prune perineum syndrome (PPS) is a poorly recognized clinical entity. Only four cases have been published in the literature, the first being described in 1979 by Peeden et al^1^, the second in 1983 by Saad et al^2^, the third in 2016 by Lopes et al^3^, and the fourth in 2020 by Berjeaut et al^4^. Noteworthy, only in the two most recent reported cases, the patient reached childhood. PPS is characterized by both sacrococcygeal spine and perineal malformation, so as absent or hypoplastic genitalia, being the most typical finding a large “prune-like” perineal mass, giving its known definition. This report aims not only to describe the fifth case of PPS, but a newly associated feature, the cystic dilatation of the terminal ventricle.

## Case report

The patient was born from a healthy first-time mother, with no familial history of congenital malformation, at the 37th week of a twin pregnancy, being the 2nd twin of cesarean delivery. Her birth weight was 2,705 g, cephalic perimeter 36 cm, thoracic perimeter 30 cm, length 45 cm, 1st- and 5th-minute Apgar 9 and 10, respectively. Mother and newborn blood types were B positive.

**Figure 1.**
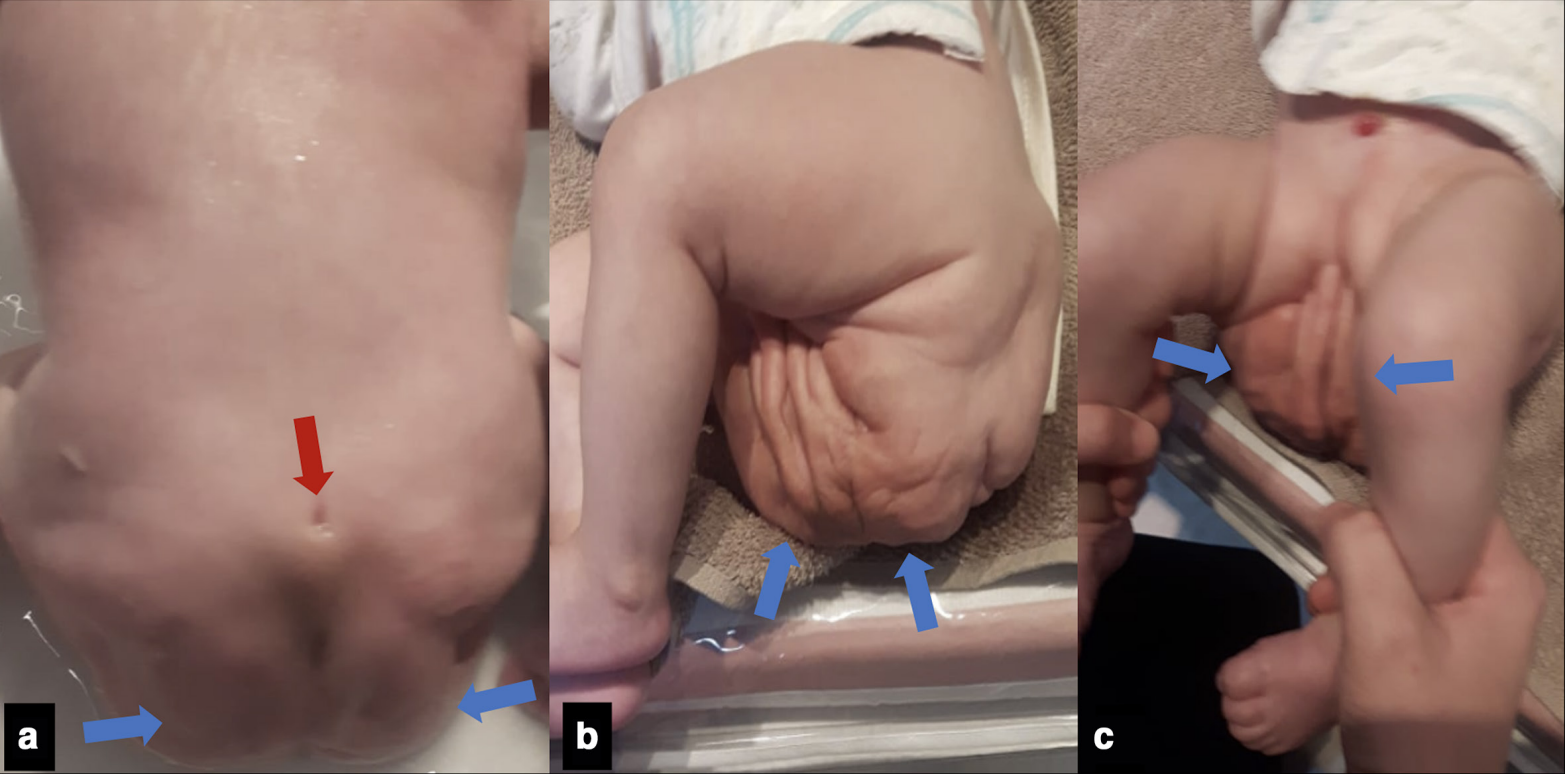
Clinical features showing (a–c) the sacral dimple with bony protuberance (red arrow) and prune-like mass (blue arrows) extending from both gluteal regions and perineum. No external genitals or anus have been characterized.

The most evident clinical finding was a large mass extending from both gluteal regions and perineum, with a tiny central orifice draining urine, without a definitive urethral meatus. No external genitals or anus were characterized. A dimple was noted on her sacral region, a few centimeters below the iliac crest line, and a bony appearance elevation was seen during spine palpation (Figure 1). There was no evidence of other abnormalities above the umbilicus. The newborn’s lower and upper limb movements were normal, and there were no immediate post-labor complications.

The other twin was born without any clinical findings or evident malformations and remained normal cognitive and growing development. There are no other siblings.

Due to the entity’s unrecognition, the newborn was observed and investigated during the first few days. The karyotype was normal (46 XX) – so as her twin sister, the echocardiogram showed a patent foramen ovale and persistence of the left superior vena cava. Spine ultrasound detected a rhombus conus medullaris termination with no masses within the spinal cord. Abdominal ultrasound detected an anterior rotation of the left kidney. No bladder or uterus has been detected. Spine radiography showed a pubic diastasis and sacrococcygeal eversion, with a bony protrusion oriented upward.

**Figure 2.**
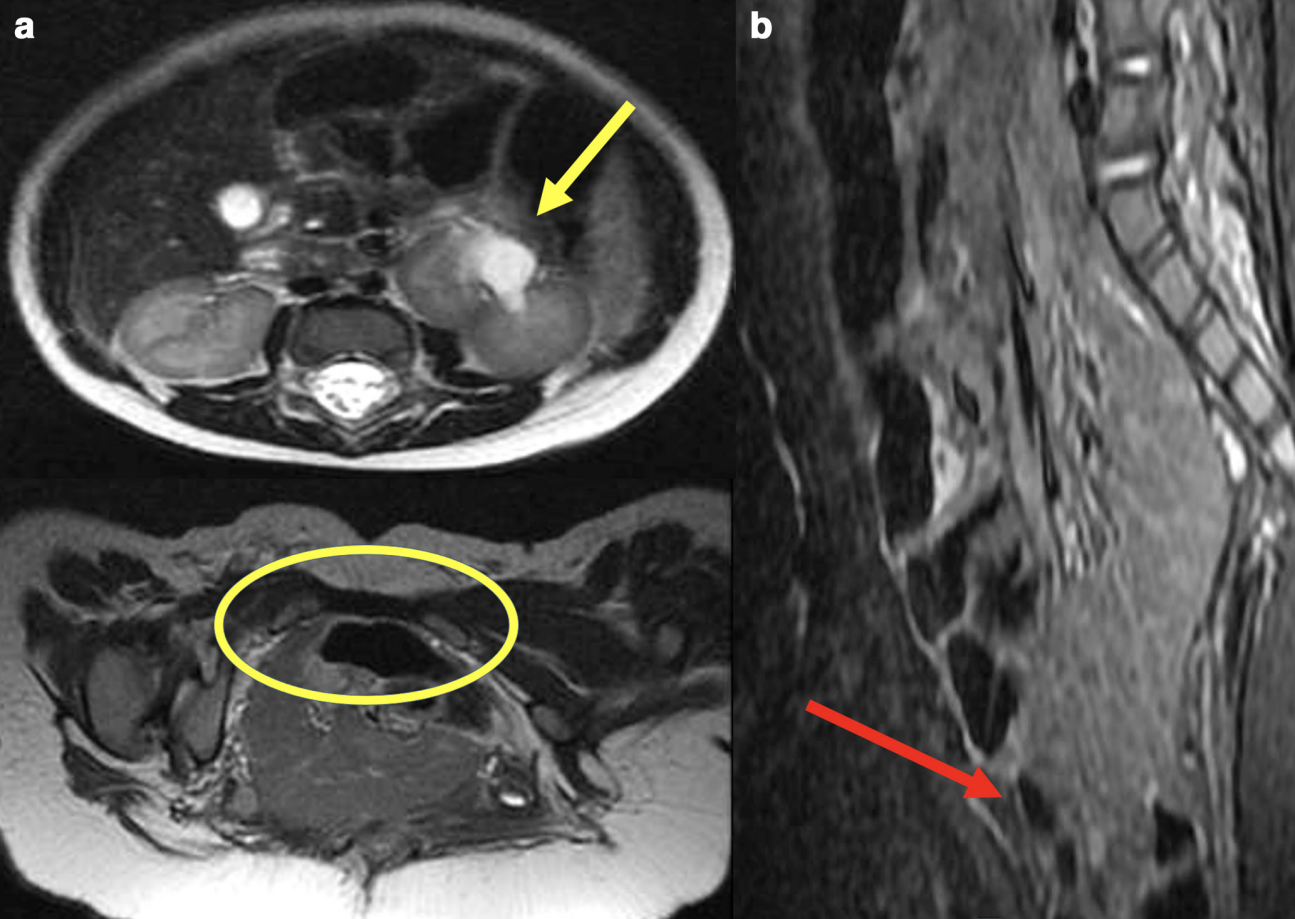
Abdominal MRI findings. Axial *T*_2_WI (a, b) imaging demonstrating the left kidney abnormal incomplete rotation with mild proximal pyelocaliceal distention (yellow arrow) and the pubic diastasis (yellow circle). Sagittal *T*_2_WI with fat suppression showing the small bowel loops inferior protrusion, contributing to the “prune-like” mass (red arrow). *T*_2_WI, *T*_2_weighted imaging

Abdominal MRI was performed in her 2nd-day hospitalization (Figure 2), showing a left kidney abnormal incomplete rotation with mild proximal pyelocaliceal distention, with no Mullerian abnormalities. Middle and distal ureters were not distended and both proximal ureters conjoined to an amorphous pelvic structure that had a urinary leak. No ureteral fusion was detected. The uterus and the urinary bladder were not characterized. A marked small intestine protrusion was seen through the perineum, in a pubic diastasis, leading to bulging and pronounced intestinal distension (Figure 2). There was no specific study for pelvic musculature.

Due to progressive abdominal dilatation and colonic obstruction caused by anus atresia, a left colostomy was performed on her 5th day of hospitalization. The patient required 2 days of mechanical ventilatory post-operative assistance and has been discharged on her 8th-day, without complications. No abnormalities were found in brain MRI.

**Figure 3.**
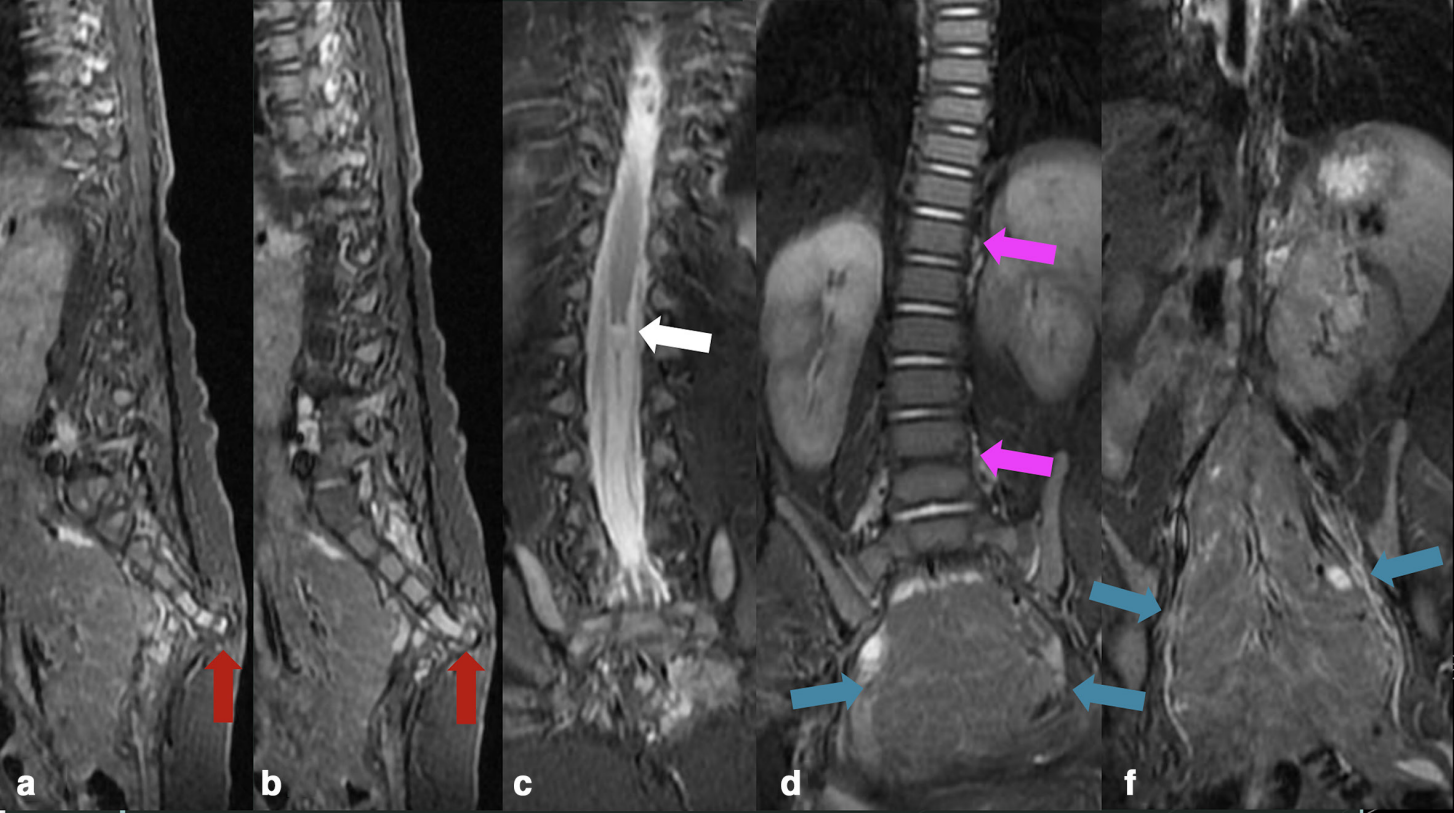
Sagittal (a, b) and Coronal (c–e) *T*_2_WI with fat suppression additionally demonstrating thoracolumbar scoliosis with right convexity (pink arrows) and the prune-like mass protruding through the perineum (blue arrows). *T*_2_WI, *T*_2_weighted imaging.

**Figure 4.**
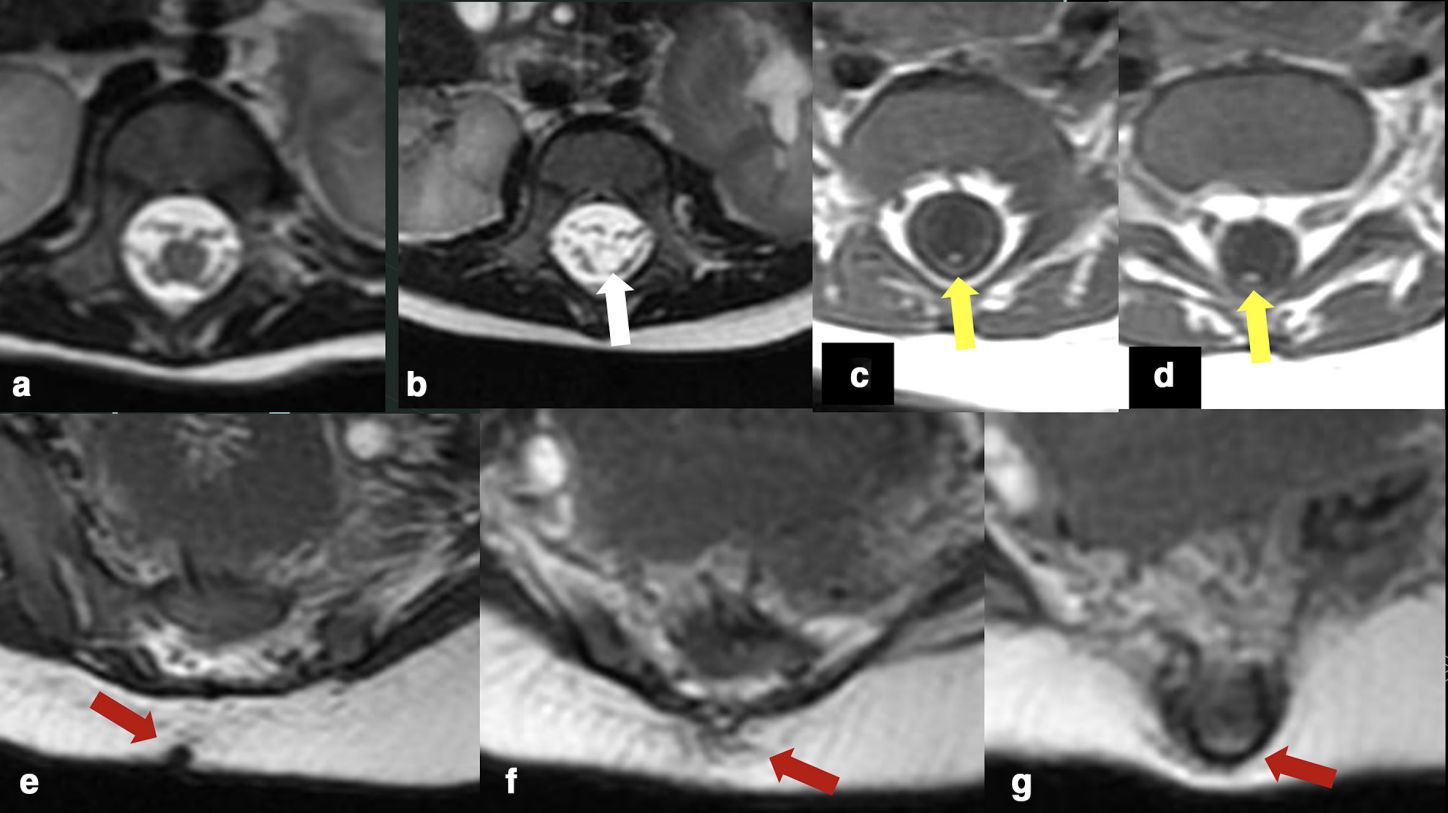
Axial *T*_2_WI (a, b) and *T*_1_WI (c–g) demonstrating the cystic dilation of the terminal ventricle (white arrow), a small lipoma of the filum terminale (yellow arrows), and a fibrous band connecting the sacral dimple with the bony protuberance. No connection with the thecal sac has been characterized. *T*_2_WI, *T*_2_weighted imaging

Spine MRI findings were consistent with developmental anomalies characterized by the anterior fusion of the L4 and L5 vertebral bodies (block vertebra), thoracolumbar scoliosis with right convexity, rectification, and posterior orientation of the sacrococcygeal elements towards the skin/subcutaneous cellular tissue (sacral eversion), where a fibrous band, part of the external “dimple” was inserted, without communication to the thecal sac (Figures 3 and 4).

**Figure 5.**
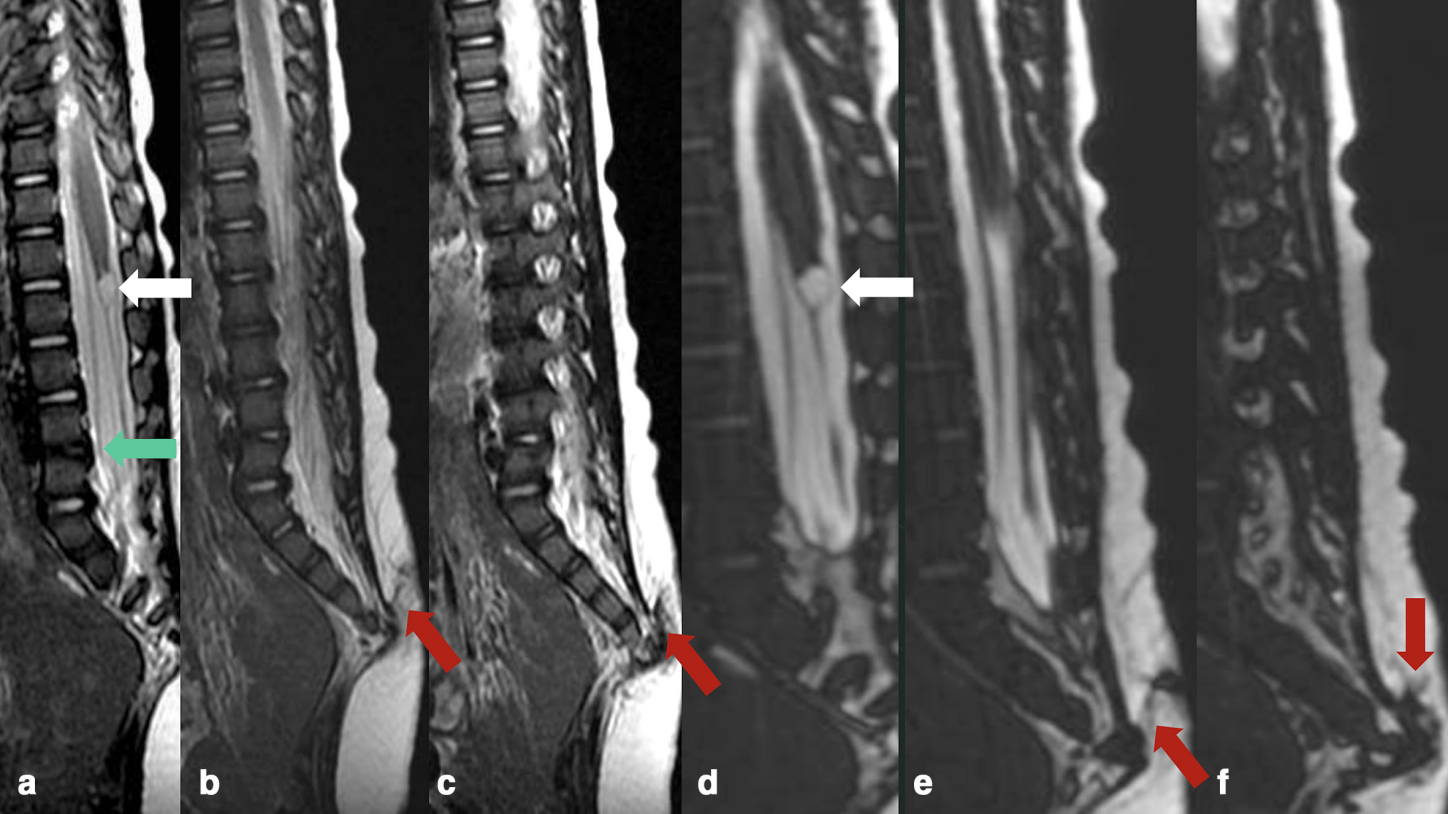
Sagittal *T*_2_WI (a–c) and Sagittal 3D-CISS (d–f) imaging demonstrating the cystic dilation of the terminal ventricle (white arrows), the anterior fusion of the L4 and L5 vertebral bodies (block vertebra) (green arrow), rectification and posterior orientation of the sacrococcygeal elements towards the skin/subcutaneous tissue (red arrows).

Additionally, there was a cystic dilatation of the terminal ventricle at the level of the distal portion of the medullary cone (T12-L1), measuring approximately 8.0 mm in its largest diameter. A small lipoma of the filum terminale was also noted (Figures 4 and 5).

Currently, the patient has 6 months of age with normal neurological and psychomotor development and remains with a colostomy bag, so as under intermittent catheterization. The next surgical step aims for urinary diversion through a continent abdominal reservoir and “prune-like” mass correction. Although pediatric surgeons have discussed this strategy, due to lack of literature evidence, it remains undefined. Due to pandemics, genetic analysis to reveal possible mutations were not done yet.

## Discussion

PPS is an extremely rare and unknown entity. A typical “key” diagnostic feature of PPS is the presence of a “prune-like” redundant perineal mass with mucous (internal) and cutaneous (external) epithelium containing malformed pelvic structures with a common fistulous ureteral pathway draining into the perineal cavity. To date, there is no connection between this entity and sex because all previous related four cases were 46 XY, differing from ours. Twin pregnancy maybe is not a risk factor either, explained by the fact that our case is the only one published in this presentation.

Lopes et al^3^ postulated an embryologic explanation for the orifice draining urine in the mass, which consists of a prenatal probable fistula rupture between the “perineal bladder” and skin before due to increased intravesical pressure. Still, no other explanations were found in the literature. As in our case, all the described patients with PPS have normal motor and cognitive development.

Misdiagnosis can be made with associated anorectal and genital tract malformations, like anal atresia-persistent cloaca, chromosomic aberrations related to anal atresia, maternal diabetes mellitus associated sacral dysgenesis, and progesterone-estrogen or progesterone hormonal pills anal–rectal-related malformations, some of which included in the VACTERL spectrum.

Terminal ventricle cyst or fifth ventricle is a fusiform ventricle, measuring 8–10 mm in length and 2–4 mm in transverse diameter located in the conus medullaris. A few relations between this finding and congenital abnormalities have been postulated, but it is currently known for its typical benign course^5,6^ Major differences are syringohydromyelia and hydromyelia.

Four cases of PPS have been described to date with no consensus in therapeutic strategies. The third case was first managed by doing a continent cutaneous ileal neobladder. However, 4 months after surgery, the patient developed an acute obstructive abdomen, followed by septic shock and death.^3^ The fourth case provided relevant information and strategies aiming at malformations correction and life quality improvement, consisting first in an osteotomy, followed by definitive colostomy, cystoscopy evaluation, cystoplasty, perineal and abdominal correction with mesh, bilateral orchiopexy, treatment of the vesicocolonic fistula, sigmoidectomy and appendivesicostomy.^4^ The cited patient is 6 years old, walks with no assistance, and has good abdominal tonus.

## Learning points

1. PPS is an extremely rare and underrecognized entity.
2. PPS’s key diagnostic feature is the presence of a “prune-like” redundant perineal mass containing malformed pelvic structures with a common fistulous ureteral pathway draining into the perineal cavity.
3. The main differential diagnosis of this entity consists of VACTERL disease spectrum.
4. Cystic dilatation of the terminal ventricle is a new associated finding in this entity.
5. There is still no consensus in the therapeutic management of PPS, and the prognosis remains low. Still, some new strategies have been published to aim malformations correction and life quality improvement.

## Conclusion

PPS is an extremely rare disease, and its correct recognition is vital to avoid misdiagnosis with other malformations, with emphasis on VACTERL. Our case is the fifth in the literature and the first with an associated cystic dilation of the terminal ventricle. Due to lack of experience and possible underrecognition of this entity, we strongly suggest future research focusing on both physiopathological mechanisms and associations, as well as patient’s management. Although cystic dilatation of the terminal ventricle could be an incidental finding on the neonatal lumbar spine, it can be present in this syndrome and may add in future reports and identification of this condition.
